# Identification of the key stages for sex determination in the silkworm, *Bombyx mori*

**DOI:** 10.1007/s00427-013-0461-9

**Published:** 2013-12-18

**Authors:** Hiroki Sakai, Fugaku Aoki, Masataka G. Suzuki

**Affiliations:** Division of Biological Sciences, Department of Integrated Biosciences, Graduate School of Frontier Sciences, The University of Tokyo, 302 Bioscience-Bldg, 5-1-5 Kashiwanoha, Kashiwa-shi, Chiba-ken 277-8562 Japan

**Keywords:** *Doublesex*, Sex determination, *Bombyx mori*, Insulin-like growth factor II mRNA-binding protein, *Feminizer*

## Abstract

In general, the master switch gene for sex determination is expressed for a limited period during the early embryonic stage. To increase our understanding of the sex determination mechanism in *Bombyx mori*, it is important to understand when sex determination takes place. To examine the key stages for sex determination in this insect, we focused on the expression patterns of *Bmdsx* (a double-switch gene in the sex determination cascade of *B. mori*) and *BmIMP* (a gene expressed specifically in males involved in male-specific splicing of *Bmdsx*). Reverse transcription PCR (RT-PCR) analysis revealed that male-type *Bmdsx* expression was observed in females at 27 and 29 h after oviposition (hao), and finally disappeared at 32 hao. Moreover, *BmIMP* mRNA was also expressed in these females, and its expression level was comparable to that of the male-type *Bmdsx* mRNA. These results demonstrated that female embryos before 32 hao can show male-type expression of *Bmdsx* and *BmIMP*, suggesting that sex determination occurs between 29 and 32 hao, which correspond to the developmental stages from the head lobe differentiation to spoon-shaped embryo stages. This also suggests that the master switch gene for sex determination of *B. mori* is expressed in females during this period and represses the male-specific mode of expression in sex-determining genes.

## Introduction

*Bmdsx*, an orthologue of *doublesex* (*dsx*), displays sex-specific expression in various tissues in *B. mori*. The male- and female-specific splice isoforms of *Bmdsx* encode male-specific BmDSX protein (BmDSXM) and female-specific DSX proteins (BmDSXF), respectively (Suzuki et al. 2001). Ectopic expression of BmDSXM in females resulted in abnormal differentiation of certain female-specific genital organs and caused partial male differentiation in female genitalia (Suzuki et al. 2005). In contrast, BmDSXF functions as a positive regulator of the hexameric storage protein SP1 and vitellogenin genes, which are expressed predominantly in females (Suzuki et al. 2003). Therefore, *Bmdsx* functions as a double-switch gene at the bottom of the sex determination cascade of *B. mori*. In *Drosophila melanogaster*, female-specific splicing of *dsx* requires TRA, TRA-2, and the exonic splicing enhancer elements located within the *dsx* fourth exon (Nagoshi and Baker 1990). However, a *tra* orthologue has not been identified in the *Bombyx* genome (Mita et al. 2004). Moreover, RNAi knockdown of the *Bombyx* orthologue of *tra-2* has no influence on sex-specific splicing of *Bmdsx* pre-mRNA (Suzuki et al. 2012). These facts strongly suggest that the mechanism of sex-specific splicing at the *doublesex* gene differs between *D. melanogaster* and *B. mori*. In *B. mori*, the splicing inhibitor BmPSI and the *Bombyx mori* insulin-like growth factor II mRNA-binding protein (BmIMP) are involved in the regulation of male-specific splicing of *Bmdsx* (Suzuki et al. 2010). *BmIMP* is localized on the Z chromosome and is male-specifically expressed in various tissues. Therefore, *BmIMP* is thought to play a role in the sex determination cascade in *B. mori*. Genetic studies have revealed that female sex in *B. mori* is determined by the presence of a dominant feminizing factor, *Feminizer* (*Fem*), on the W chromosome (Tanaka 1916). However, *Fem* has not yet been identified and its function remains unknown. In general, the master switch gene for sex determination is expressed for a limited period during the early embryonic stage. For example, the expression of *Sry*, which is the master switch gene for mammalian sex determination, is regulated in a strictly time-dependent manner. *Sry* is expressed between approximately E10.5 and E12.5, with a peak at E11.5 (Kashimada and Koopman 2010). *Sex-lethal* (*Sxl*), which is the master switch gene at the top of the sex determination cascade in *Drosophila* (Salz et al. 1989), and *DM-W*, which is a probable sex-determining gene in *Xenopus laevis*, are both expressed within a narrow time frame (Yoshimoto et al. 2008). Therefore, determining when sex determination occurs may facilitate identification of the master switch gene for sex determination in *B. mori*.

In the present study, expression patterns of *Bmdsx* and *BmIMP* at different embryonic stages were investigated by reverse transcription PCR (RT-PCR) to determine the key stages for sex determination in *B. mori*. We found that sex determination of *B. mori* occurs between 29 and 32 hao. In contrast to our expectations, female embryos at 27 and 29 hao expressed male-specific *Bmdsx* and *BmIMP*, as well as female-specific *Bmdsx*. Our results support the concept that the master switch gene for sex determination in *B. mori* is expressed in females between 29 and 32 hao and represses the male-specific mode of expression in sex-determining genes. To our knowledge, this is the first report of the identification of the sex determination stage in lepidopteran insects.

## Materials and methods

### Silkworm strains

We used two silkworm strains; namely, *pnd-w1,* a non-diaposed and white egg strain maintained in the National Institute of Agrobiological Sciences, and *S-1,* a non-diaposed and sex-limited black egg strain. For this strain, the pink-eyed white egg (*pe*/*pe*) is male (Z/Z) while the black-eyed black egg (+^*pe*^/*pe*) is female (Z/W) and homozygous for *pnd*. This strain has a large chromosome composed of the W chromosome, chromosome 2 bearing the *p* ^*B*^ gene, and chromosome 5 bearing the +^*pe*^ gene (Tanaka et al. 2000). Therefore, eggs of this sex-limited strain, in which females have a genotype of T (W; 2, 5) *pB* +^*pe*^, *pe* (egg, black; larva, black) and males have a genotype of *pe* (egg, white; larva, white), were examined. The developing eggs were incubated at 25 °C. Larvae were reared on an artificial diet (Nihon Nosan) at approximately 25 °C.

### Sample preparation

Male and female moths were mated for 3 h at 25 °C. The female moths were then kept at 5 °C overnight. To collect oviposited eggs, female moths were transferred to the rearing room and allowed to lay eggs every 0.5 h at 25 °C. These eggs were stored at 25 °C.

### Microscopic observation of eggs

Eggs were observed using a stereomicroscope (SZX12, Olympus). Microscopic images were photographed using a DP50-CU (Olympus).

### RNA extraction and RT-PCR

Total RNA was extracted from each egg using Isogen (Nippon Gene), as described previously (Suzuki et al. 2012). RT-PCR reactions were performed as described previously (Suzuki et al. 2012). The female- and male-specific *Bmdsx* isoforms were amplified with primers FDSX-F2 (5′-CGC CTT ACC GCA GAC AGG CAG-3′) and FDSX-R4 (5′-GCG CAG TGT CGT CGC TAC AAG G-3′) under the following conditions: 94 °C for 2 min, 35 cycles of 98 °C for 10 s, 57 °C for 30 s, and 72 °C for 1 min, followed by 72 °C for 2 min. *BmIMP* cDNA was amplified as described previously (Suzuki et al. 2010). Amplification of GAPDH cDNA as a positive control for the RT-PCR reaction was performed using primers GAPDH-F (5′-GCC GCA TTG GCC GTT TGG TGC-3′) and GAPDH-R (5′-CAT GAA CAG TAG TCA TCA AGC-3′) under the following conditions: 94 °C for 2 min, 26 cycles of 98 °C for 10 s, 57 °C for 30 s, and 72 °C for 1 min, followed by 72 °C for 2 min.

### Quantitative real-time RT-PCR

Quantitative real-time RT-PCR assays were performed using SYBR Premix Ex Taq II (TaKaRa Bio, Inc.) on a Thermal Cycler Dice Real Time System (TaKaRa Bio, Inc.) according to the manufacturer’s instructions. BmIMPE7-F (5′-ATG CGG GAA GAA GGT TTT ATG-3′) and BmIMP-R (5′-TCA TCC CGC CTC AGA CGA TTG-3′) were used for the quantification of the *BmIMP* gene expression. GAPDH-F and GAPDH-R were used for the quantification of *GAPDH* as an internal standard. The threshold cycle (CT) value was normalized with the CT value of the *GAPDH* gene using Multiplate RQ software (TaKaRa Bio, Inc.).

## Results and discussion

**Fig. 1:**
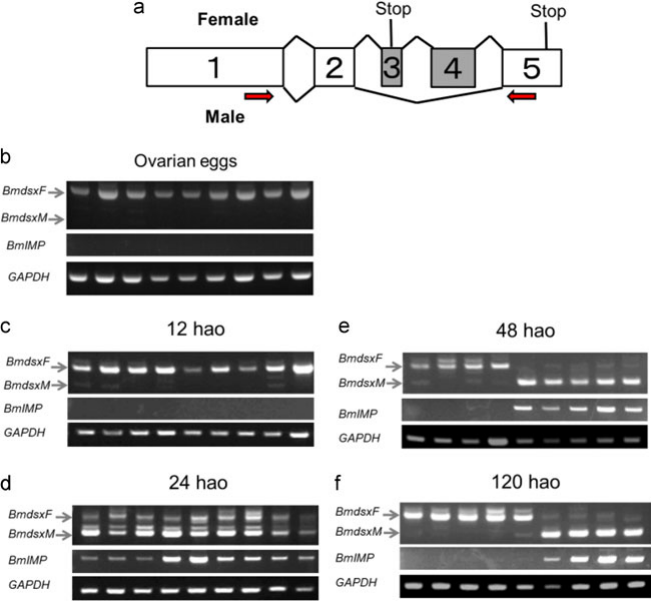
Expression patterns of *Bmdsx* and *BmIMP* during the early developmental stages. **a** Schematic diagram of alternative splicing in *Bmdsx* pre-mRNA. *Boxes* represent exons. The *gray region* indicates the female-specific exons. The *numbers in the diagram* represent exon labels. *V-shaped lines above* (inclusion of alternative exons) and *below* (skipping of alternative exons) the diagram represents the endogenous *Bmdsx* splice variants observed in females and males. Stop codons are indicated by “*stop*.” The *arrows* indicate the approximate location of the primers used for RT-PCR in **b**–**f**. These primers can amplify several female-specific isoforms other than the *BmdsxF* isoform, as described previously (Shukla et al. 2011). To investigate developmental changes in the expression patterns of *Bmdsx* and *BmIMP*, RT-PCR was performed with total RNA extracted from ovarian eggs (**b**), eggs at 12 (**c**), 24 (**d**), 48 (**e**), and 120 (**f**) h after oviposition (*hao*). Nine eggs at each stage were subjected to the analysis. *Bottom panel* shows the results of RT-PCR amplification of the GAPDH transcript, which served as a positive control for the RT-PCR reaction. PCR products were analyzed on a 1 % agarose gel. *Arrows to the left* of the gel refer to position *BmdsxF* and *BmdsxM*

To determine the key stages for sex determination, we investigated developmental changes in the expression patterns of *Bmdsx* and *BmIMP* by RT-PCR analysis with total RNA extracted from ovarian eggs and eggs at 12, 24, 48, and 120 h after oviposition (hao). Molecular sexing by PCR using W chromosome-specific primers is often used to discriminate female eggs from male eggs. However, our previous study demonstrated that molecular sexing is not applicable to eggs at early developmental stages as more than 30 % have a significant number of polar-body-derived cells (Sakai et al. 2013). For this reason, RT-PCR (Fig. 1) was performed without sexing of the examined eggs. Nine eggs at each stage were subjected to the analysis. Ovarian eggs and eggs at 12 hao expressed the female-specific *Bmdsx* (*BmdsxF*) isoform, but did not express *BmIMP* (Fig. 1b, c). Both *BmdsxF* and the male-specific *Bmdsx* isoform (*BmdsxM*) were expressed in all examined eggs at 24 hao (Fig. 1d). These eggs also expressed *BmIMP*. In contrast, two types of expression patterns were observed in individuals at 48 hao; some individuals expressed *BmdsxF* without *BmIMP* expression, while others expressed both *BmdsxM* and *BmIMP* (Fig. 1e). It is possible that the former group was female and the latter group was male. These results suggested that the sex determination occurred between 24 and 48 hao.

**Fig. 2:**
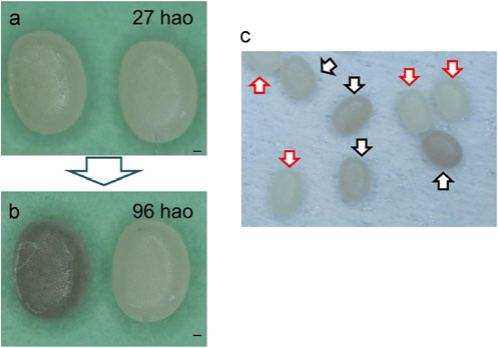
Pigmentation of eggs obtained from the sex-limited strain S-1. In the sex-limited strain, S-1, pigmented (*black*) eggs become female (ZW) while nonpigmented (*white*) eggs become male. Female eggs were separated from male eggs according to the extent of egg pigmentation. **a** Eggs obtained from S-1 strain at 27 hao. The left egg started to pigment. **b** The same egg as shown in (**a**) displayed complete pigmentation at 96 hao. **c** Eggs at 32 hao. *Black arrows* indicate incompletely pigmented eggs. *Red arrows* indicate nonpigmented eggs. The batch (eggs from a single pair mating) presented in this figure contained incompletely pigmented eggs and nonpigmented eggs at a ratio of 1:1

To further explore the key stages for sex determination, eggs obtained from a sex-limited strain, *S-1*, were subjected to the same analysis as described in Fig. 1. Eggs of the sex-limited strain displayed differences in pigmentation according to the sex; nonpigmented (white) eggs were male, while pigmented (black) eggs were female (see “Materials and methods”). We found that several eggs from *S-1* strain started to pigment at 27 hao under our experimental conditions (Fig. 2a). A total of 30 eggs with the same extent of incomplete pigmentation as in Fig. 2a were collected at 29 hao and kept at 25 °C. All these eggs completely pigmented before 96 hao (Fig. 2b). This suggested that eggs with incomplete pigmentation at 27–29 hao can be considered genetically ZW females. However, that does not necessarily mean that nonpigmented eggs are all males since most female eggs did not start to pigment during those stages. Some batches at 32 hao (eggs from a single pair mating) contained incompletely pigmented eggs and nonpigmented eggs at a ratio of 1:1 (Fig. 2c), which is equal to the sex ratio in *B. mori*. Therefore, we regarded these nonpigmented eggs as genetically ZZ males. Based on these findings, female eggs were collected after 27 hao, while male eggs were collected after 32 hao in this study.

**Fig. 3:**
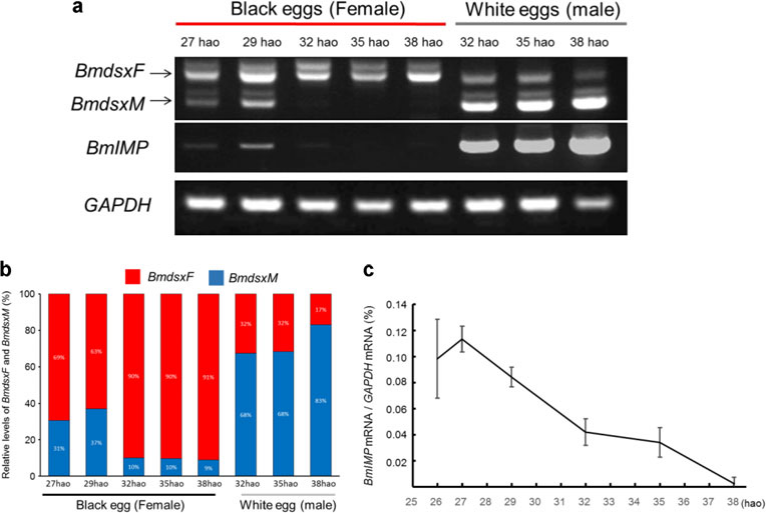
Expression patterns of *Bmdsx* and *BmIMP* in female and male eggs during early developmental stages. **a** The same RT-PCR analysis as described in Fig. 1 was performed using pooled total RNAs isolated from either 10 female eggs or 10 male eggs at each indicated stage. The *bottom panel* shows the results of RT-PCR amplifications of the GAPDH transcript, which served as a positive control for the RT-PCR reaction. PCR products were analyzed on a 1 % agarose gel. *Arrows to the left* of the gel refer to the positions of *BmdsxF* and *BmdsxM.* **b** Quantification of the RT-PCR analysis shown in panel **a**. *BmdsxF* and *BmdsxM* relative to sum of these expressions. Product DNAs were quantified using ImageJ 1.47v software (Wayne Rasband, National Institutes of Health, USA: http://rsb.info.nih.gov/ij/). **c** Expression profile of *BmIMP.* RT-qPCR was performed using total RNA extracted from an egg and three eggs were examined in each experiment. *Error bars* represent standard errors. In this experiment, the female eggs started to pigment at 26 hao and the peak of *BmIMP* expression was different from the results indicated in panel **a**

The same RT-PCR analysis as described in Fig. 1 was performed using pooled total RNA isolated from either 10 female eggs or 10 male eggs. As shown in Fig. 3a, females at all examined stages highly expressed the *BmdsxF* isoform. Surprisingly, *BmdsxM* and *BmIMP* were markedly expressed in females at 27 and 29 hao. The expression level of the *BmdsxM* isoform correlated with that of *BmIMP*. The expression of *BmdsxM* and *BmIMP* mRNAs were dramatically diminished in female eggs after 32 hao (Fig. 3a, b). The reduction in the expression level of *BmIMP* mRNA in female eggs was also confirmed by the RT-qPCR (Fig. 3c). However, males at all examined stages highly expressed *BmdsxM* and *BmIMP*. These results demonstrated that female embryos before 32 hao can induce male-specific expression of *Bmdsx* and *BmIMP*, suggesting that sex determination occurs between 29 and 32 hao. The embryos at 29 hao and 32 hao are expected to be in the head lobe differentiation stage (stage 5) and spoon-shaped embryo stage (stage 6), respectively (Takami and Kitazawa 1960). Therefore, the sexual fate of *B. mori* would be determined during the early stages of spoon-shaped embryos.

Our results also suggested that the expression level of *BmIMP* was comparable to that of *BmdsxM* (Figs. 1 and 3a). BmIMP physically interacts in vitro with BmPSI, which is expressed in both sexes, and the transfection of cDNAs encoding these two proteins into female cultured cells induces male-specific splicing of *Bmdsx* (Suzuki et al. 2010). Therefore, it is possible that the expression of *BmdsxM* in females is mediated by the expression of *BmIMP*. This supports that *BmIMP* plays a crucial role in male sex determination. The function of *BmIMP* in sex determination is currently being explored.

**Fig. 4:**
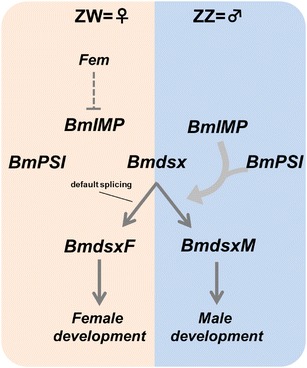
A plausible model of sex determination in *B. mori*. The sexual fate of *B. mori* is determined genetically; ZW for females and ZZ for males (Tanaka 1916). The putative female-determining gene, *Fem*, is located on the W chromosome. Both ZZ and ZW individuals express *BmIMP* before the sex determination stage (during the early spoon-shaped embryo stage). *Fem* is expressed during the sex determination stage and then directly or indirectly represses the expression of *BmIMP* in females. In the absence of BmIMP protein, *BmdsxF* is predominantly expressed as the *BmdsxF* splice variant is produced by default splicing (Suzuki et al. 2001). BmDSXF protein promotes female development. In males, zygotic expression of *BmIMP* can be maintained during the developmental stages due to the absence of *Fem*. The BmIMP protein, together with *BmPSI*, induces male-specific splicing of *Bmdsx*, leading to the production of BmDSXM protein. BmDSXM promotes male development

The sexual fate of *B. mori* is determined genetically; ZW for females and ZZ for males (Tanaka 1916). A putative female-determining gene, *Feminizer* (*Fem*), is thought to be located on the W chromosome. However, *Fem* has not yet been identified and the function of this gene remains unknown. As described above, our results indicate that female embryos in early developmental stages are capable of inducing male-specific expression of *Bmdsx* and *BmIMP*. This suggests that the master switch gene for sex determination of *B. mori* is expressed in females during the early developmental stages (during the early spoon-shaped embryo stage) and represses the male-specific mode of expression in sex-determining genes, including *Bmdsx* and *BmIMP* (Fig. 4). We are attempting to identify a W chromosome-specific gene that is expressed exclusively in females during the sex determination stage to identify the dominant female-determining gene, *Fem*.
